# Tau PET imaging in neurodegenerative tauopathies—still a challenge

**DOI:** 10.1038/s41380-018-0342-8

**Published:** 2019-01-11

**Authors:** Antoine Leuzy, Konstantinos Chiotis, Laetitia Lemoine, Per-Göran Gillberg, Ove Almkvist, Elena Rodriguez-Vieitez, Agneta Nordberg

**Affiliations:** 1 Division of Clinical Geriatrics, Center for Alzheimer Research, Department of Neurobiology, Care Sciences and Society, Karolinska Institutet, Stockholm, Sweden; 2 Theme Neurology, Karolinska University Hospital, Stockholm, Sweden; 3 Department of Psychology, Stockholm University, Stockholm, Sweden; 4 Theme Aging, Karolinska University Hospital, Stockholm, Sweden

**Keywords:** Neuroscience, Molecular biology

## Abstract

The accumulation of pathological misfolded tau is a feature common to a collective of neurodegenerative disorders known as tauopathies, of which Alzheimer’s disease (AD) is the most common. Related tauopathies include progressive supranuclear palsy (PSP), corticobasal syndrome (CBS), Down’s syndrome (DS), Parkinson’s disease (PD), and dementia with Lewy bodies (DLB). Investigation of the role of tau pathology in the onset and progression of these disorders is now possible due the recent advent of tau-specific ligands for use with positron emission tomography (PET), including first- (e.g., [^18^F]THK5317, [^18^F]THK5351, [^18^F]AV1451, and [^11^C]PBB3) and second-generation compounds [namely [^18^F]MK-6240, [^18^F]RO-948 (previously referred to as [^18^F]RO69558948), [^18^F]PI-2620, [^18^F]GTP1, [^18^F]PM-PBB3, and [^18^F]JNJ64349311 ([^18^F]JNJ311) and its derivative [^18^F]JNJ-067)]. In this review we describe and discuss findings from in vitro and in vivo studies using both initial and new tau ligands, including their relation to biomarkers for amyloid-β and neurodegeneration, and cognitive findings. Lastly, methodological considerations for the quantification of in vivo ligand binding are addressed, along with potential future applications of tau PET, including therapeutic trials.

## Introduction

The misfolding and accumulation of proteins in the brain is a feature common to a range of neurodegenerative disorders, including a collective characterized by the accumulation of pathological tau. Referred to as tauopathies, the accurate identification of these disorders is a challenge clinically, particularly early on in the symptomatic course, because of overlapping clinical phenotypes. The advent of tau-specific ligands for use with positron emission tomography (PET), however, has now made it possible to investigate tau deposition at early stages of several neurodegenerative disorders, including Alzheimer’s disease (AD), progressive supranuclear palsy (PSP), corticobasal degeneration (CBD), and related conditions such as Down’s syndrome (DS), Parkinson’s disease (PD), and dementia with Lewy bodies (DLB). In addition to assisting in the accurate and early differential diagnosis of these disorders, tau imaging may also prove to be of value in the study of tau in the aging brain, in naturalistic studies as a predictor of future cognitive decline, and in therapeutic trials, both for subject selection and as a surrogate outcome measure. In this review, we describe and discuss in vitro and in vivo findings in the brain from investigation of various tau ligands, other biomarkers and cognitive measures, and with respect to methodological considerations tied to the quantification of tau PET.

## Tau deposits

**Fig. 1:**
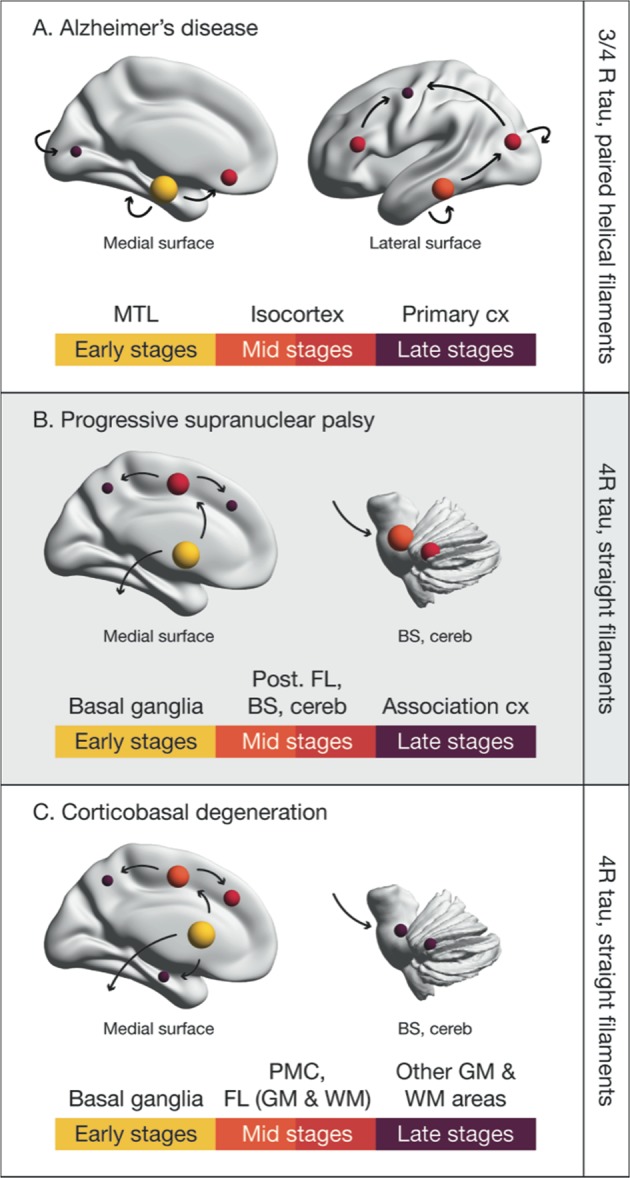
Spreading schemes for tau pathology in Alzheimer’s disease (AD), progressive supranuclear palsy (PSP), and corticobasal degeneration (CBD). The color coding and the size of the spheres distinguishes between brain areas affected at early (yellow; large size), middle (shades of red; mid size) and late (purple; small size) stages of tau propagation. The scheme for AD is adapted from Braak and Braak [60]. The scheme for PSP is adapted from Williams et al. [18]. The scheme for CBD was inspired by the neuropathological studies performed by Forman et al. [19], Kouri et al. [20], and Ling et al. [21]. The label at the right side of each panel indicates the predominant tau isoform affected and the type of tau fibrils formed in AD, PSP, or CBD. For a more detailed description of the neuropathological findings, please refer to Fig. 4. BS brainstem, cereb cerebellum, cx cortex, FL frontal lobe, GM gray matter, MTL medial temporal lobe, PMC primary motor cortex, post. FL posterior frontal lobe, WM white matter

A phosphoprotein involved in the stabilization of microtubules, tau is natively unfolded, with six isoforms divided into two functional groups based on the number of repeats [three (3R) or four (4R)] of the microtubule-binding domain [1]. Tau is normally phosphorylated, but hyperphosphorylation weakens its binding to the microtubules and increases its cytosolic levels [2]; following a migration from axonal to somatodendritic compartments, hyperphosphorylated tau assembles into protofibrils [3]. These assemblies are classified as straight, twisted or paired helical filaments (PHFs), based on the absence or periodicity of twists; they are found in neurons, astrocytes, and oligodendroglia [4]. Tau aggregates can assume a range of ultrastructural polymorphisms on the basis of isoform predominance and post-translational modifications [5–9]. Although the mechanisms leading to tau pathology are as yet unclear, experimental evidence implicates abnormalities in kinase and phosphatase activity [10], as well as chronic cerebral hypoperfusion [11], in the hyperphosphorylation of tau; once hyperphosphorylated, decreased microtubule binding results in the increased release of soluble tau species [12]. Transfer of such species between cells has then been shown to occur via synaptic [13, 14] and nonsynaptic pathways [12, 15], resulting in seeding and the induction of tau aggregation in recipient cells. The accumulation of tau pathology, however, has been postulated to follow stereotypical spatiotemporal patterns. Figure 1 illustrates the tau spreading schemes in AD, PSP, and CBD, as suggested by pathology studies [16–21], although much uncertainty in the exact spreading patterns of tau remains, especially in non-AD tauopathies given the relative paucity of neuropathological data in those diseases in comparison to AD, as well as their neuropathological heterogeneity. According to pathological investigations of tau at autopsy, in vivo tau PET imaging is expected to shed further light on the time course of tau accumulation in relation to other biomarkers and clinical symptomatology.

## First- and second-generation tau tracers

**Fig. 2:**
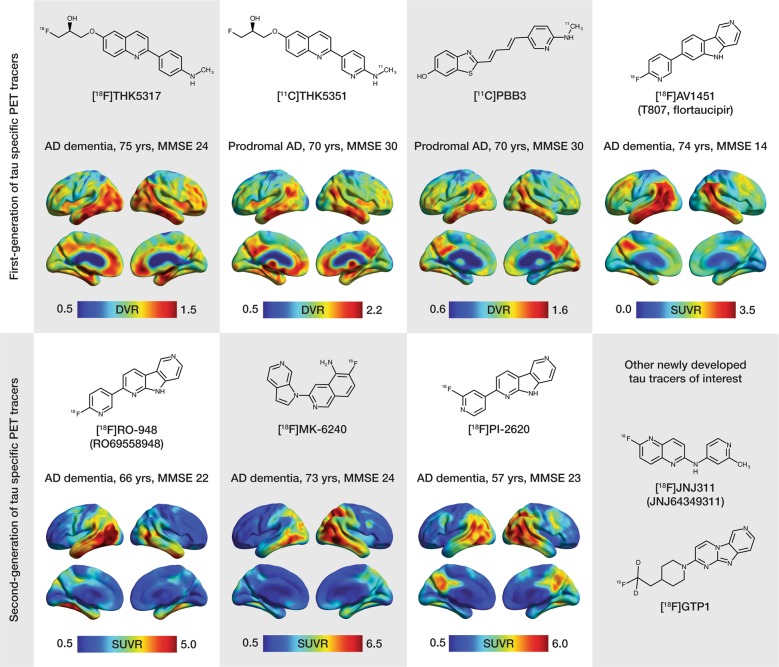
Chemical structures and representative uptake images in amyloid-β-positive Alzheimer’s disease (AD) patients using selected first- (upper portion of the figure) and second- (lower portion of the figure) generation tau PET tracers. The characteristics in terms of clinical research diagnosis, age and mini mental-state examination (MMSE) scores are presented for each patient above the respective image. For the creation of parametric images for all tracers, areas of the cerebellar cortex were used as reference. The [^18^F]THK5317, [^11^C]THK5351 and [^11^C]PBB3 images derive from studies performed at Karolinska Institutet, Center for Alzheimer Research [35, 153]. The [^18^F]AV1451 image is courtesy of the Alzheimer’s disease neuroimaging initiative (ADNI). The [^18^F]RO-948 image is courtesy of Ruben Smith and Oskar Hansson (Lund University, Lund, Sweden). The [^18^F]MK-6240 and the [^18^F]PI-2620 images are courtesy of Vincent Doré, Christopher Rowe and Victor Villemagne (University of Melbourne, Victoria, Australia) and Andrew Stephens and Mathias Berndt (Piramal Imaging GmbH, Berlin, Germany), respectively. Different scales were used to better illustrate the regional distribution pattern of binding for each tracer, due to between-patient differences as well as due to the different PET acquisition parameters and quantification methods that were applied for each tracer. Though direct comparison is complicated by these differences, one can observe the preferential binding of the first- and second-generation tracers in AD-relevant areas of the temporal lobes, and the broader dynamic range among second-generation tracers. DVR distribution volume ratio, SUVR standardized uptake value ratio

Despite the challenges inherent in imaging tau [22, 23], remarkable progress has been made over the past few years; [^18^F]FDDNP was the first PET tracer developed to target tau but due to lack of specificity and selectivity in vivo [24], a new set of tau PET tracers was synthetized. The so-called first-generation ligands (e.g., [^18^F]THK5317, [^18^F]THK5351, [^18^F]AV1451 (also known as [^18^]T-807, [^18^F]flortaucipir), and [^11^C]PBB3) have been used extensively in research studies, with second-generation compounds [namely [^18^F]MK-6240, [^18^F]RO-948 (previously referred to as [^18^F]RO69558948), [^18^F]PI-2620, [^18^F]GTP1, [^18^F]PM-PBB3, and [^18^F]JNJ64349311 ([^18^F]JNJ311), and its derivative [^18^F]JNJ-067] now entering the field [25–31]. Although the first-generation was promising, some off-target binding led to the optimization of the binding properties and development of this second-generation of tau tracers followed. Figure 2 shows the chemical structures of the available tau tracers. It is worth noting the differences in the chemical structures of the first-generation tracers. With the aim of developing tracers with better specificity, as discussed below, some of the second-generation tracers were based on the structures of existing tracers (i.e., [^18^F]RO-948, [^18^F]PI-2620), but others have relatively different structures (i.e., [^18^F]MK-6240, [^18^F]JNJ311).

### In vitro studies: the what and where of tracer binding

Extensive in vitro characterization of first-generation tau tracers, using autoradiography on both frozen and paraffin sections of AD brains in comparison with immunostaining of tau deposits and other hallmarks, was carried out to determine their binding properties. THK5117, THK5351, PBB3, and AV1451 (with the lack of labels here, and throughout the text, referring to the molecule itself, whether labeled with carbon-11, fluorine-18, tritium, or unlabeled in competition binding or autoradiography studies) all bind to intracellular and extracellular neurofibrillary tangles (NFTs), as well as to ghost tangles and neuritic plaques [32] (Table 1). Contradictory findings, however, have been reported for binding to pretangles: Ono et al. [33] reported no binding for both PBB3 and AV1451 while, more recently, Wren et al. [34] showed binding of THK5117 and T726, the fluorescent analogue of AV1451. Finally, inter- as well as intra-case differences were reported in the study of Wren et al. This study emphasized the fact that more detailed and complete investigation of the binding characteristics of the different tau tracers are needed in order to be able to draw conclusions as to their relationship to varied tau characteristics, including the type of tau involved (3R vs. 4R), fibril isoform (paired helical vs. straight vs. twisted), maturity level of the tau deposit (pretangle, mature tangle, and ghost tangle), as well as cell type affected (neurons vs. glia).

**Table 1:** Binding characteristics of selected first- and second-generation tau tracers in in vitro studies

		Ki/IC50	Kd	Bmax	On-target	Off-target
First-generation tau PET tracers	PBB3	Ki_1_: 1.3; 5.9 Ki_2_: 23.5	Ki_1_:2.5; Kd_2_:100	Bmax_1_: 25 nM; Bmax_2_: 300 nM	NFTs, neuropil threads, neuritic plaques; pick bodies, tau inclusion in PiD, PSP, CBD	Dense core amyloid plaques; diffuse amyloid-β- deposits, astrocytic plaques
	AV1451	Ki_1_: 0.3; 3.3 Ki_2_: 97.2	0.63–3.72; 15/14.6	15–119.7	PHFs tau, pretangles, mature tangles, neuritic and primitive plaques (to a limited extent)	Dense core amyloid-β plaques; melanin containing structure, lipofuscin containing structures, mineralized structures, MAO-A, MAO-B
	THK5117	Ki_1_: 0.001; 0.0005 Ki_2_: 10.5–27.4 Ki_3_: 750-800	Kd_1_: 2.2–3.1; Kd_2_: 23.6–34.6/Kd: 5.19–11.5/5.2	Bmax_1_: 250 Bmax_2_: 1226–1416; 338	Pretangles, PHF tau, NFsT, neuritic plaques, argyrophillic grains, argyrophilic threads, globose tangles	MAO-B
	THK5351	Ki_1_: 0.1 pM Ki_2_: 16	2.9 mol/l; Kd_1_: 5.6; Kd_2_: 1	368; Bmax_1_: 76, Bmax_2_: 40	NFTs, thread like structures in white matter, tufted astrocytes	MAO-B
Second-generation tau PET tracers	MK-6240	Ki:0.36	0.14–0.38	7–93.4 nM	NFTs	No off-target binding to MAO-A, MAO-B
	RO-948	IC50: 18.5		9.7	NFTs, neuropil threads	Extensive screening shows no off-target binding
	RO1643	IC50: 10		5.5	NFTs, neuropil threads	Extensive screening shows no off-target binding
	RO4693	IC50: 5.5		5.9	NFTs, neuropil threads	Extensive screening shows no off-target binding
	PI-2620	IC50: 1.8			3R tau from Pick, 4R from PSP	No off-target binding to MAO-A, MAO-B
	JNJ311	Ki = 8			PHF tau, neuritic plaques	
	JNJ-067		2.4		NFTs	No off-target binding to MAO-A, MAO-B
	GTP1		14.9 ± 4.3			No off-target binding to MAO-A, MAO-B
	PM-PBB3		<10		Tau aggregates in AD and PSP	
	AM-PBB3		<10		Tau aggregates in AD and PSP	No off-target binding in basal ganglia and thalamus
References		[30–33, 123, 149, 152, 239–242]	[25, 26, 32, 34, 123, 142, 149, 152, 239, 240, 243–245]	[25, 26, 32, 123, 142, 149, 152, 239, 241]	[26, 30–34, 78, 111, 112, 118, 142, 149, 150, 152, 239, 241, 242, 244–247]	[26, 32, 33, 111, 112, 123, 146, 149, 241, 242, 245, 247]

### In vivo tau PET

#### Ageing and AD

##### First-generation tau tracers

Tau imaging in cognitively normal (CN) elderly individuals, using [^18^F]THK tracers [35, 36], [^11^C]PBB3 [26], and [^18^F]AV1451 [37–48] has consistently shown ligand retention to be largely restricted to the medial temporal lobe (MTL), with cortical findings variable and relatively low [49], or absent altogether [50]. This pattern of MTL binding is consistent with the neuropathological literature [51] and may reflect an age-related process of tau accumulation in this region [52], so-called primary age-related tauopathy [53–56], which has been shown to result in hippocampal atrophy and mild amnestic deficits that are amyloid-β-independent [54, 57, 58].

Patients with AD have significantly higher levels of tau tracer retention than CN individuals [25, 26, 35–37, 39, 40, 42, 44, 59] including in the inferior lateral temporal, posterior cingulate, and lateral parietal regions, with binding  matching the known regional deposition of tau pathology reported in histopathological studies [51, 60]. Fig. 2 shows sample brain images using different tau tracers for in vivo investigation of patients with AD. Other studies comparing CN individuals with patients with mild cognitive impairment (MCI) showed differences in binding restricted to MTL regions [39, 40], as well as lateral temporal and parietal areas when examining only amyloid-β-positive MCI [35]. In a recent large-scale study that examined the discriminative accuracy of [^18^F]AV1451, for the separation of both MCI due to AD and AD dementia from other neurodegenerative disorders, sensitivity, and specificity estimates were highest using temporal and temporoparietal *meta*-regions of interest (ROIs) [61]. Group separation was lower in MCI due to AD, however, owing to less pronounced tracer binding, as compared to AD dementia [61]. In patients with atypical AD presentations, the spatial pattern of [^18^F]AV1451 retention closely matched that in brain regions underlying the different clinical phenotypes [44, 62–67] and had topographical overlap with reduced glucose metabolism patterns [44, 65–67]. In the few studies addressing longitudinal tau PET [68–71], observable increases in tau PET were reported, although significant findings were confined to the subject level in one study [69]. In the largest of these studies [70], tau accumulation rates were found to be rather uniform across brain regions, arguing against the notion that the build-up of tau pathology progresses in a stepwise manner, as suggested by histopathological studies. However, the discrepancy between histopathological and in vivo PET findings could be associated with varied factors relating to the lack of extensive validation of the existing tracers (see section, "Postmortem validation of non-AD tau binding"), or to the fact that this tau staging scheme is based on cross-sectional autopsy data from different brains, thus amounting to an extrapolation only.

##### Second-generation tau tracers

In the studies using second-generation tau tracers published to date, which are few [72–74], binding of [^18^F]RO-948 was elevated in medial temporal areas as well as more broadly throughout the cortex, including in the precuneus/posterior cingulate, lateral parietal and occipital lobes, and prefrontal cortex, in four AD patients in comparison to six healthy controls [72]. AD patients showed higher [^18^F]RO-948 retention in the temporal (hippocampus, entorhinal cortex, parahippocampus, inferior, and middle gyri), medial frontal, and inferior parietal cortices in comparison to older controls. There was increased tracer retention in these regions, in addition to the supramarginal gyrus, precuneus and lateral occipital lobe, in a subset of three AD patients who underwent follow-up [^18^F]RO-948 PET (median = 21 months). Using [^18^F]MK-6240, binding patterns consistent with Braak staging of neurofibrillary tau were observed across an amyloid-β-positive cohort comprising older controls (including those exhibiting cognitive decline), two MCI patients and seven AD patients [73]. Lohith et al. [74] reported similar high [^18^F]MK-6240 binding in neocortical and medial temporal brain regions in four AD patients, two MCI patients, and four healthy controls. Preliminary in vivo evidence supports binding of [^18^F]PI-2620 predominantly in the temporal cortex in AD patients (unpublished data). No in vivo human data have been presented for the tracers of the JNJ family yet. Sample images of patients with AD who underwent PET imaging with selected second-generation tau tracers are shown in Fig. 2.

### Primary and other tauopathies

#### Primary tauopathies

**Fig. 3:**
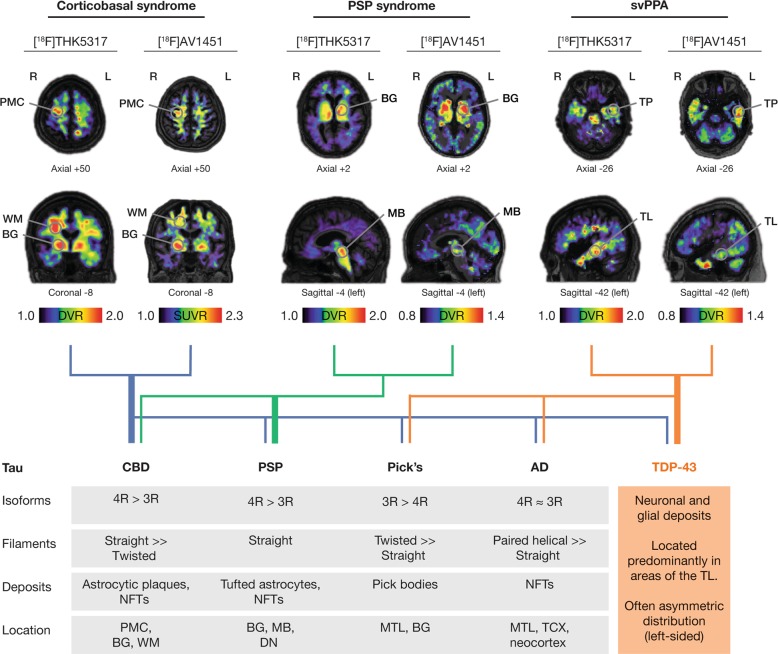
Representative [^18^F]THK5317 and [^18^F]AV1451 images of amyloid-β-negative patients with clinical diagnoses of corticobasal syndrome, progressive supranuclear palsy (PSP) syndrome, and a semantic variant of primary progressive aphasia (svPPA) (upper portion of the figure), and a tree diagram (mid portion of the figure) illustrating the *poor* correlation between the clinical diagnoses of corticobasal syndrome, PSP syndrome, and svPPA with pathological confirmation of the presence of tau [black font; corticobasal degeneration (CBD), PSP, Pick’s disease, Alzheimer’s disease (AD)], and TDP-43 (orange font) pathologies. The areas with high-tracer uptake are indicated with circles. The thickness of the strings (OR lines) in the diagram illustrates the approximate strength of the clinicopathological correlations [156, 237]. The lower portion of the figure shows the neuropathological and biochemical characteristics of tau pathology seen across a number of tauopathies, as well as some typical neuropathological characteristics of TDP-43 pathology. Of note, different patients were scanned for each tau PET tracer, although one can observe apparent similarities in the regional distribution of tracer uptake for [^18^F]THK5317 and [^18^F]AV1451 in the patients with the same clinical diagnosis. The patients with a clinical diagnosis of corticobasal and PSP syndromes show high binding of both tracers in relevant areas, in agreement with the expected regional distribution of CBD and PSP pathologies, respectively. Interestingly, high binding with both tracers is observed even in patients with a clinical diagnosis of svPPA, a syndrome which is not primarily associated with the presence of tau, but rather TDP-43 pathology. For the creation of parametric images for all tracers, areas of the cerebellar cortex were used as reference. The [^18^F]THK5317 images derive from studies performed at Karolinska Institutet, Center for Alzheimer Research [35]. The [^18^F]AV1451 images of the patient with corticobasal syndrome is courtesy of Chul Hyoung Lyoo (Yonsei University College of Medicine, Seoul, South Korea) [80], while the [^18^F]AV1451 images of the patients with PSP syndrome and svPPA are courtesy of Simon P. Jones and James Rowe (University of Cambridge, Cambridge, UK) [82, 138]. Different scales were used to better illustrate the regional distribution pattern of binding for each tracer, due to between-patient differences as well as due to the different PET acquisition parameters and quantification that were applied for each tracer or even within the same tracer between different laboratories for [^18^F]AV1451. BG basal ganglia, DN dentate nucleus, DVR distribution volume ratio, MB midbrain, PMC primary motor cortex, SUVR standardized uptake value ratio, TL temporal lobe, TP temporal pole, TCX temporal cortex, WM white matter

Although most tau PET studies have been performed in patients with AD, tau imaging should be of clinical value in other more rare neurodegenerative conditions associated with tau pathology such as CBS and PSP [75]. In addition to NFTs composed of PHFs in neurons, as in AD, these neuropathological entities also exhibit tau in glial cells. These deposits consist mainly of 4R tau, which in turn forms straight tau filaments that are located mainly in subcortical nuclei. As can be observed in Fig. 3, however, there is substantial clinical and neuropathological overlap between these tauopathies, and with other syndromes associated with cognitive impairment, including AD. This renders clinical diagnosis challenging, particularly when combined with the absence of reliable disease-specific biomarkers [76, 77].

So far, PET imaging solely with first-generation tau tracers has been carried out in primary tauopathies. [^18^F]AV1451, tracers of the [^18^F]THK family, and [^11^C]PBB3 have, in single cases or groups of patients with a clinical diagnosis in the CBS/PSP spectrum, shown the regional pattern of tau pathology expected in these diseases with relatively good discrimination from healthy volunteers [19, 26, 35, 78–88]. However, given the fact that many of the regions of interest in CBS and PSP largely coincide with areas showing off-target binding [i.e., to monoamine oxidase (MAO)-B in the basal ganglia], there is a wide overlap in the load of tracer binding in those regions across diagnostic groups, especially for [^18^F]AV1451, which is the tracer that has been tested in larger patient samples [81, 84, 89]. Longitudinal tau imaging in individual patients with clinical CBS has shown an increase in tracer binding with disease progression [69, 90], while tracer binding in PSP patients correlates well with clinical scores of functional impairment [86, 91], although the latter finding has so far not been replicated in other, larger patient samples with [^18^F]AV1451 [81, 83].

Chronic traumatic encephalopathy (CTE) is also a neurodegenerative disease associated with the presence of tau pathology (3R and 4R tau) and is secondary to repeated head injuries [92]. Tau PET imaging ([^18^F]AV1451) has been reported so far only in two cases with clinical diagnosis of CTE, where a somewhat increased tracer binding was found, although with a different regional pattern of binding between patients [93, 94]. The limited evidence available illustrates that extensive work is required for determining the usefulness of tau PET imaging in CTE.

#### Other tauopathies

DS, PD, and DLB have been associated with the presence of tau deposits, primarily of the AD type [95–98]. Preliminary evidence in DS with the tracer [^18^F]AV1451 indicates that the binding pattern in amyloid-β-positive patients resembles that observed in AD, increasing with progression of the disease [99]. On the other hand, the [^18^F]AV1451 binding in PD and DLB has been largely variable, often producing inconsistencies between studies and overlap with CN controls [81, 100–105]. These findings are in agreement with the available postmortem data, which indicate a varying degree of tau pathology in these diseases; although the neuropathological presence of tau has been associated with specific clinical phenotypes of DLB/PD in some studies [106, 107], the role of tau in the development of these diseases is not yet clear [108–110]. A note of caution is advised in the interpretation of PD and DLB results, however, given that the affinity of the existing tau tracers for the hallmark pathology observed in these disorders, α-synuclein, is not fully understood (for a detailed description, please refer to the section “Tracer specificity for tau”).

#### Postmortem validation of non-AD tau binding

As described above, in vitro studies performed on AD brain tissue have supported binding to PHF-tau [32]. Most in vitro autoradiography studies in non-AD involved direct comparison with tau immunostaining, and showed that AV1451 was binding with relatively low, if any, affinity to the straight tau filaments of CBS and PSP [111–114]. These findings are in clear contrast to in vivo observations, which raises uncertainty about whether the observed in vivo signal derives from binding to tau or other targets (i.e. nonspecific binding). To add to this uncertainty, there were conflicting results regarding the presence of a correlation between in vivo [^18^F]AV1451 binding and postmortem tau load for patients with CBS or PSP who had [^18^F]AV1451 imaging and neuropathological assessment of tau burden; [^18^F]AV1451 PET identified in vivo areas with postmortem high tau in some brain areas and patients but not others, which probably indicates a reduced sensitivity of the tracer to non-AD tau [81, 113, 115–117]. In contrast, in vitro staining and/or autoradiography studies have highlighted the ability of PBB3 and tracers of the THK family to bind to some extent to CBS or PSP tau deposits, in agreement with in vivo findings [26, 33, 78, 118]; this appears to have been confirmed in the first patient with ante-mortem [^18^F]THK5351 imaging and postmortem assessment of tau pathology [119]. Furthermore, in vitro evidence suggests that PBB3 may have a higher affinity for non-AD tau than AV1451 [33], and preliminary in vivo data suggest that selected THK tracers may be better able to detect non-AD pathology than AV1451 [120]. Overall, in vitro and in vivo evidence (cross-sectional and longitudinal), although somewhat preliminary, suggest that tracers of the THK family, primarily, and, secondarily, [^11^C]PBB3, may be better suited than [^18^F]AV1451 for tracking non-AD tau pathology [26, 35, 113, 118–121], although the latter remains to be confirmed by ante-/post-mortem findings in CBS and PSP.

A direct comparison of in vivo and in vitro results, however, is not straightforward due the different methodological parameters of these techniques. In vitro binding studies performed with homogenates and autoradiography, for instance, can give different results caused by the washing away of extracellular proteins in homogenate-based analyses, but not in autoradiography tissue sections. In addition, results can be widely influenced by the concentration of ethanol in the incubation buffer, the purity of tritiated compounds (which can decrease over time in contrast to carbon-11 and fluorine-18 labeled PET compounds, which are custom synthesized on the day of the experiment), and choice of paraffin (including pretreatment, heat and fixation of the tissue) vs. frozen sections [122]. Indeed, a recent study highlighted the importance of experimental design for in vitro studies, showing that the concentration of ethanol used determined concordance with in vivo findings [122]. As a further example, when Harada et al. performed their initial evaluation of the THK tracers on paraffin sections, evidence of potential off-target binding of the tracer to MAO-B was not detected (personal communication, Ryuichi Harada). Conversely, when the same evaluation was performed on frozen tissue, a competition of the tracer with a MAO-B inhibitor was apparent, as discussed below, in agreement with in vivo studies [35, 123, 124].

As recently pointed out in a commentary by Klunk [125], there is still a lack of extensive experimental and clinical validation of different tau PET tracers. Some of the differences might be due to tracer metabolism and lipophilicity, a case in point being [^11^C]PBB3, where the estimation of brain retention is complicated by the presence of a brain penetrant radioactive metabolite [26,126,127]. There is also assumptions pertaining to the relationship between semi-quantitative estimates and findings from arterial input modeling that likely do not always hold (e.g., the commonly used late frame quantification of tracers and the actual measures of tracer binding, show only moderate correlation for tracers of the THK family [128] and a nonlinear relationship for [^18^F]AV1451) [129, 130]. Together, these elements limit, to some extent, the interpretation of existing cross-sectional and longitudinal data. Finally, in addition to the fact that in vivo and in vitro studies, such as autoradiography and immunostaining, are likely capturing different stages of pathology—with postmortem studies limited to a single time point, most often at the end stage of the disease—they cannot be directly compared as antibodies are targeting specific epitope and phosphorylation sites, whereas PET tracers bind to varied molecular sites. We do believe that in vitro studies are necessary, however, and that experimental efforts need to be devoted to better understanding which aspects may contribute to discrepancies between in vivo and in vitro evidence for current tau tracers.

#### Tracer specificity for tau

##### Off-target binding to non-tau protein deposits

The existing tau tracers are small molecules and although we think of them as specific to tau itself, their specificity is actually based on the structure of the tau β-sheet that is adopted when tau forms the different kinds of fibrils in AD or other tauopathies. However, this structure is not unique to tau but is also present in the fibrils formed by amyloid-β, TDP-43, α-synuclein, etc [131]. As an example, [^18^F]FDDNP, which was the first tracer injected in vivo with affinity for tau pathology [132–134], and was developed several years before the reviewed first and second-generation tau tracers, showed a lack of specificity for tau over other β-sheet structures [135, 136], which limited the interest in its further development. Such specificity issues should serve as a didactic example, indicating that the need for extensive validation of the binding characteristics of the tau tracers currently under development. Interestingly, the first in vitro studies of the first-generation tau tracers highlighted a selective binding to tau rather than to other fibrils [111, 112, 118], but increasing evidence now indicates that these tracers can potentially bind to other protein deposits. For instance, both [^18^F]AV1451 and fluorine-18 THK ligands have demonstrated a similar characteristic regional binding pattern in vivo in PET studies of a series of patients with a semantic variant of primary progressive aphasia (svPPA), a clinical syndrome associated predominantly with TDP-43 pathology rather than tau pathology (Fig. 3) [86, 137–139]. Furthermore, [^18^F]AV1451 and [^11^C]PBB3 have shown in vivo binding in patients expected to have α-synuclein deposits [140, 141], which is in agreement with some more recent in vitro studies, at least for [^11^C]PBB3 – so far, however, autoradiography results do not confirm a similar in vitro binding of THK5351 and AV1451 to α-synuclein deposits [33, 111, 142, 143].

While observations with [^18^F]AV1451 and [^11^C]PBB3 remain to be validated in ante-/post-mortem studies, they raise concerns that the different tau tracers might bind in vivo to multiple β-sheet structures, a finding that would limit their clinical utility in discriminating between both different protein deposits—which regularly coexist in different proportions in the neurodegenerating brain irrespective of the driving pathology [144]—and different proteinopathies. Furthermore, binding of the tracers to multiple β-sheet structures could limit the interpretation of studies investigating the association between those different co-existent pathologies (i.e., amyloid-β, tau, α-synuclein, and TDP-43) in patients with cognitive complaints, and especially those looking into the association between tau and amyloid-β in AD (studies discussed below), where both pathologies are involved and are often colocalized.

##### Off-target binding to other molecular structures

**Fig. 4:**
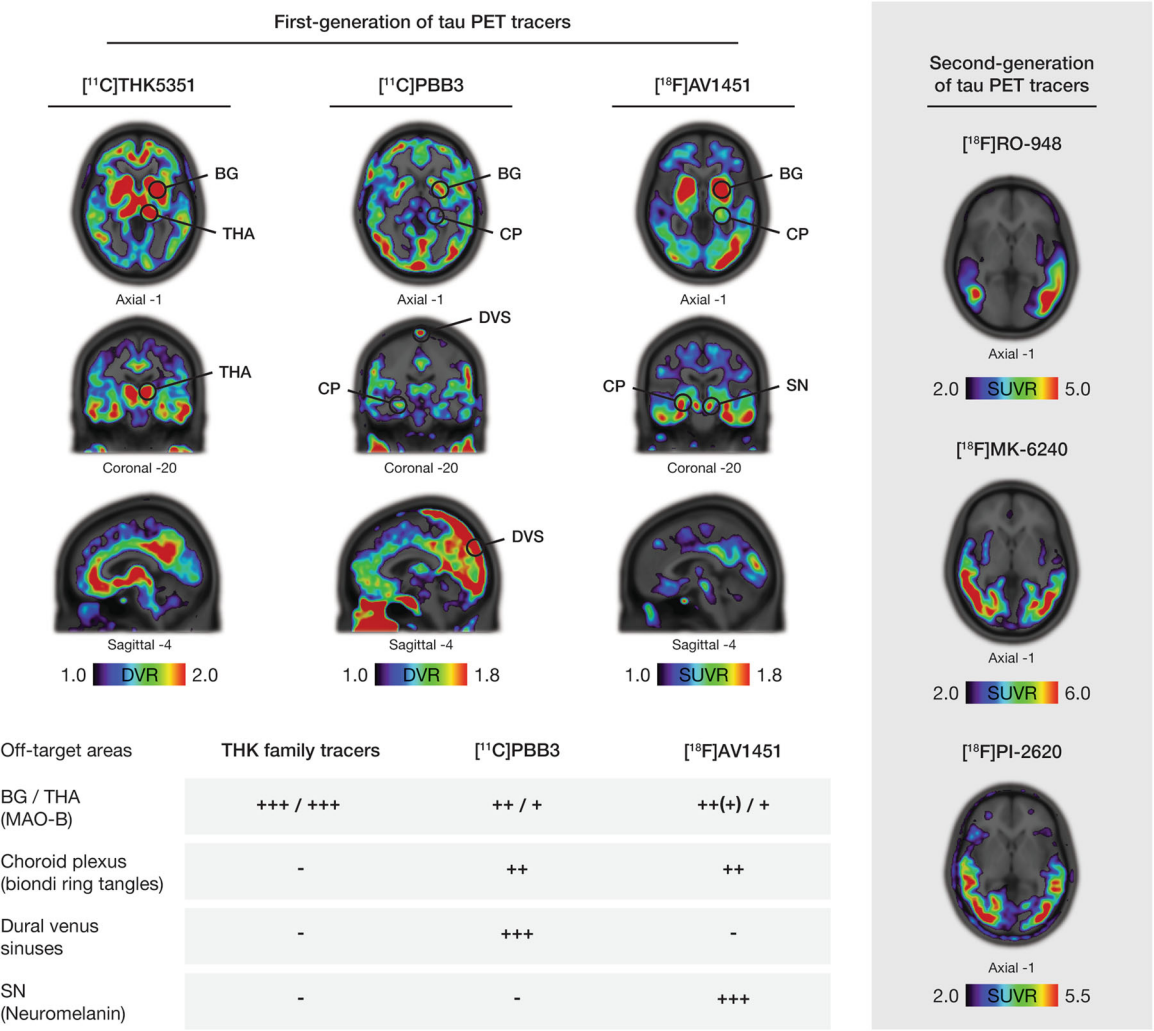
Off-target binding of selected first- (left portion of the figure) and second (right portion of the figure) -generation tau PET ligands. Images are taken from amyloid-β-positive Alzheimer’s disease (AD) patients; research clinical diagnosis, age, and mini-mental state examination results are as follows: [^18^F]THK5351, prodromal AD, 70, 30; (Karolinska Institutet, Center for Alzheimer Research); [35] [^11^C]PBB3 prodromal AD, 53, 27 (Karolinska Institutet, Center for Alzheimer Research); [153] [^18^F]AV1451, AD dementia, 79, 22 (image courtesy of ADNI); [^18^F]RO-948, AD dementia, 66, 22 (image courtesy of Oskar Hansson and the Swedish BioFINDER study); [^18^F]MK-6240, AD dementia, 73, 24 and [^18^F]PI-2620, AD dementia, 57, 23 (images courtesy of Christopher Rowe and Victor Villemagne). The main areas of known off-target binding for the second-generation tau tracers are marked with circles. The load of this off-target binding across the different tracers is graded in a semiquantitative manner in a table in the lower portion of the figure. Specific non-tau targets for this binding have been reported as briefly named in parentheses and illustrated in detail in Table 1. The wider dynamic range of the second-generation tau tracers can be seen as well as the lower binding in the MAO-B rich basal ganglia (BG) and thalamus (THA). Different scales were used to better illustrate the regional distribution pattern of binding for each tracer, due to between-patient differences as well as due to the different PET acquisition parameters and quantification that were applied for each tracer. Other reported off-target binding areas for the second-generation tau tracers are not presented in this figure [27, 29]. Areas of the cerebellar cortex were used as reference for creating parametric images for all the tracers. CP choroid plexus, DVS dural venous sinuses, SN substantia nigra, BG basal ganglia, DN dentate nucleus, DVR distribution volume ratio, MB midbrain, NFTs neurofibrillary tangles, PMC primary motor cortex, R repeats of the microtubule-binding domain, SUVR standardized uptake value ratio, TL temporal lobe, TP temporal pole, TCX temporal cortex, WM white matter

Off-target binding to molecular structures other than tau protein deposits discussed above have been reported for first-generation tracers, in particular to MAO-B enzyme in the basal ganglia (Fig. 4), a finding that has been validated in vitro, at least for the THK tracers and [^18^F]AV1451 [124, 145]. This could limit the clinical validity of these PET tracers, at least in diseases where tau deposition is expected in areas with a high load of MAO-B (e.g., CBS and PSP); the potential implications of this with respect to the interpretation of isocortical signal, however, remains unclear [146]. Although off-target binding of second-generation tracers has yet to be described in detail, they are thought to be associated with a lower, or no, signal in MAO-B-rich areas (Fig. 4).

An off-target signal has been reported for first-generation tau tracers in vascular structures (choroid plexus for [^11^C]PBB3 and [^18^F]AV1451, and the dural venous sinuses for [^11^C]PBB3, attributable to biondi ring tangles, hemosiderin, melanocytes, etc. [32, 147]), with this potentially affecting accurate quantification of tracer binding in the relevant areas (e.g., MTL and isocortex) [72, 73]. [^18^F]AV1451 and its derivatives from the second-generation (e.g., [^18^F]RO-948) have been associated with binding to neuromelanin, however, which could indicate that these tracers would be useful in PD because of the characteristic loss of neuromelanin-rich neurons of the substantia nigra [72, 104, 105, 147].

##### Comparison of tau tracer binding in silico, in vitro, and in vivo

**Fig. 5:**
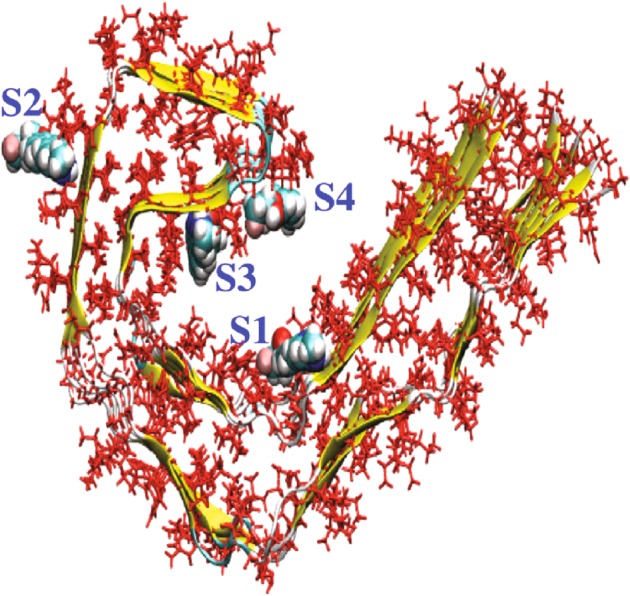
Representation of a tau protofibril, showing four high-affinity binding sites (core sites: 1, 3, and 4; surface site, 2) for tau PET ligands, as determined via in silico modeling. With the exception of [^18^F]T808, all the studied tracers ([^18^F]AV1451, [^18^F]FDDNP, [^11^C]PBB3, [^18^F]THK5105, [^18^F]THK523, [^18^F]THK5351, [^18^F]THK5117, [^18^F]MK-6240, [^18^F]RO-948, and [^18^F]JNJ311) have shown significant binding to these four sites. On the basis of molecular docking scores, however, tracers such as [^18^F]FDDNP, [^18^F]THK5351, [^18^F]RO6955, and [^18^F]MK-6240 bind preferentially to the core sites, while [^11^C]PBB3 and [^18^F]THK523 bind preferentially to site 2. Certain ligands, moreover, such as [^18^F]THK5317 and [^18^F]JNJ311, show similar binding affinities to several sites. Adapted with permission from American Chemical Society Publications, Murugan et al. [151]. (ACS Chem Neurosci. 2018. Copyright 2018)

The recent publication of the cryo-electron microscopy structure of tau filaments derived from AD patients [148] has opened a new path for designing tau PET tracers and investigating the different binding sites. Predicted binding affinities are in the nanomolar range, which is in agreement with certain experimental inhibition constant data reported for these tracers [26, 27, 142, 149, 150]. Further, using in silico modeling, Murugan et al. [151] have shown four different high-affinity binding sites on the tau protofibril (Fig. 5). AV1451 and MK-6240 appear to bind to site 1 with higher binding affinity, results that are in line with those of Hostetler et al. [149], who suggested that they could bind to a similar binding site. The THK5351 tracer binds strongly to sites 1 and 3, with site 3 being the preferred site. Results from Murugan et al. [151] suggest that PBB3 binds to the 4 sites with similar affinity, while the other tracers appear to have one or two main binding sites. Further studies are needed to compare binding data from in silico modeling of tau fibrils with data from in vitro and in vivo binding studies in the human brain. In silico computer modeling may provide new tau tracers with favorable properties for PET.

In vitro binding assays using tau fibrils or human brain homogenate and human brain sections have demonstrated that the tau tracers have good binding properties in the nanomolar range (Table 1) and suggested several binding sites for first-generation PET tau tracers. There were at least three binding sites for THK5117 [152], and at least two for PBB3 [33]. A head-to-head comparison of THK (5117, the racemic form of THK5317, and 5351) compounds, PBB3 and AV1451 in postmortem AD brain tissue showed an interaction between AV1451 and THK5351, while PBB3 showed distinct binding properties [123]. A study by Ono et al. suggested that PBB3 can bind 4R tau (CBD/PSP), while AV1451 cannot, despite both showing similar binding in AD [33]. A direct comparison of MK-6240 and AV1451 showed that they most probably bind to the same site [149]. The tau tracers showed several binding sites, one with very high affinity (pM), another with intermediate Kd values between 5 and 20 nM (probably the one that is observed in PET studies), and one with low affinity (Kd > 100 nM). More in vitro characterization is needed to better understand these different binding sites, as well as their relationship to binding  patterns seen with different tau tracers in vivo.

In vivo, the tau tracers of the first generation are distributed similarly when injected into patients with AD, as discussed above (Fig. 2). However, when tracers from the THK family, [^18^F]AV1451 and [^11^C]PBB3 where injected into the same individuals in head-to-head designs, a number of substantial differences in their tracer characteristics, as well as in their relationships with other markers of the disease, were noted [120, 153]. While the binding distribution patterns for tracers of the THK family and [^18^F]AV1451 appeared to be similar and highly correlated in terms of binding load, the regional distribution pattern for [^11^C]PBB3 was different, and there was no correlation with THK tracers for the binding load, which indicates the potentially different molecular targets for [^11^C]PBB3 relative to the targets for tracers of the THK family and [^18^F]AV1451, in agreement with in vitro and in silico simulation studies. Comparison of the binding characteristics of second-generation tracers in vivo remains to be carried out.

#### Tau PET in relation to other biomarkers

##### A/T/N: a staging scheme for AD biomarkers

In the light of recent scientific advances, a biomarker-driven research framework has been proposed [154] to update and unify the diagnostic criteria for AD recommended in 2011 by the National Institute on Aging and Alzheimer’s Association (NIA-AA) [115, 155–158]. According to the new so-called A/T/N (i.e., amyloid-β/Tau/Neurodegeneration) classification scheme, biomarkers can be grouped into those for amyloid-β [A; elevated amyloid-β levels, as measured by PET or low cerebrospinal fluid (CSF) amyloid-β_1–42_ or amyloid-β_1–42_/amyloid-β_1–40_ levels], abnormal tau [T; elevated tau PET or CSF phosphorylated tau (p-tau) levels], and neurodegeneration [N; hypometabolism on [^18^F]FDG PET, atrophy on magnetic resonance imaging (MRI), or elevated CSF total tau (t-tau) levels]. The A/T/N criteria thus allow staging of AD based solely on biomarkers.

**Fig. 6:**
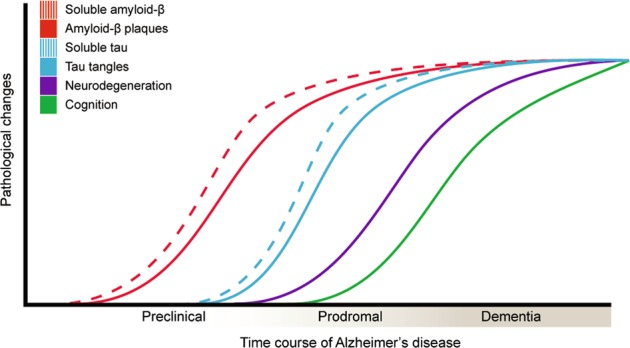
Hypothetical time course of pathological changes in Alzheimer’s disease (AD), in which biomarkers for amyloid-β become abnormal [cerebrospinal fluid (CSF) amyloid-β_1–42_ preceding PET], followed by abnormal tau (CSF p-tau preceding PET), neurodegeneration, and cognitive decline. Adapted with permission from Jack et al. [182] and 2013 [172], Nordberg [238], and McDade and Bateman [235]

Though the A/T/N framework assumes biomarker interchangeability, it recognizes a temporal delay between CSF and PET measures, with increases in soluble levels of amyloid-β and tau pathology preceding changes in PET based markers [159–167]. While this framework acknowledges the conviction shared by many in the field that amyloid-β drives the pathogenesis of AD, with pathological tau occurring as a secondary event, proximate to neurodegeneration and cognitive decline (Fig. 6), it also states that the other pathological sequences may be possible. Indeed, within the histopathological literature, both amyloid-β- [168] and tau-centric [169] outlooks have been articulated. In a third perspective, multiple, as opposed singe pathological mechanisms, (i.e., amyloid-β or tau) have been proposed to underlie the majority of dementia cases [170]. Related findings have also been described whereby tau in fact precedes amyloid-β in the brainstem and MTL [171]. Though the predominant biomarker based model of AD postulates that markers of amyloid-β are abnormal first, followed by tau and neurodegeneration [154, 172], its most recent iteration recognizes that tau may well precede amyloid-β, with these detected by immunohistology, but not current biomarkers, due its greater sensitivity [172]. As such, the partly differing time-dependent trajectories for amyloid-β and tau-tangle pathology suggested by biomarker and histopathological data may relate as much to personal viewpoints as to detection thresholds [173, 174].

##### Amyloid-β

There is now increasing evidence supporting the hypothesis that amyloid-β instigates the spread of tau beyond the MTL [37, 70, 175–177], and many in vivo imaging studies are aiming to investigate the effects of the interactive and possibly synergistic relationship between amyloid-β and tau on disease severity and progression. However, neocortical tau has been observed in a proportion of amyloid-β-negative CN individuals [177], contrary to the expected neuropathological Braak staging scheme, indicating greater heterogeneity in patterns of tau deposition than previously thought. In terms of their comparative regional distributions, though both amyloid-β and tau are elevated in neocortical regions, amyloid-β is spread diffusely throughout the neocortex, with tau pathology more selective for clinically affected brain regions [35, 38, 42, 44, 65, 67, 178–180]. When graph theory was used to investigate local and distributed network connections, PET-based measures of amyloid-β and tau were strongly correlated in the inferior-lateral temporal and entorhinal cortices, with tau accumulation in those regions being associated with widespread cortical amyloid-β [46]. Further, both showed hierarchical organization, overlapping in heteromodal association regions [181]. Indeed, while high tau has been shown within temporal regions in the absence of elevated amyloid-β levels, high-neocortical tau is associated with elevated amyloid-β levels, indicating that the deposition of amyloid-β precedes that of tau within cortical areas [178]. Studies have shown high inter-subject variability in amyloid-β and tau patterns, however, and while most AD patients present high amyloid-β and high tau [37, 42], about 15–20% of amyloid-β-positive probable AD subjects have low tau levels [178]; this may relate to low binding site concentrations or the ability of tau tracers bind varied tau conformations [178].

Overall, the accumulation of in vivo data on regional tau distribution is substantially contributing to the shedding of light onto differential biological mechanisms behind cerebral tau accumulation and spreading, in both the absence and presence of amyloid-β. The new in vivo PET data are allowing the testing of pathological spreading models that were built based only on postmortem neuropathological data [60], and are thus providing a new experimental paradigm for understanding the progression of the disease. Progress in understanding the regional and temporal relationships between amyloid-β and tau will be facilitated by further research incorporating more longitudinal data, and applying more detailed statistical approaches to interpret the complex dynamics of these proteinopathies. In particular, promising approaches may include data-driven methods, and mixed-effects model analysis that would allow for non-linear modeling of the temporal evolution of tau pathology as well as its relationship with other markers of the disease, as previously done with amyloid-β PET [172, 182]. Graph theory analysis especially in longitudinal data could shed new light on the in vivo local and distant associations between amyloid-β and tau, as well as on the temporal propagation of tau pathology especially when applied to inter-individual longitudinal data [181]. Finally, despite their more theoretical nature, mediation analyses could be used to examine potential causal relationships between pathological processes, as indexed by biomarkers [183, 184].

##### Tau (CSF P-tau)

The A/T/N classification scheme suggests that CSF p-tau and tau pathology follow in the time course of disease development after amyloid-β pathology. Several studies have now addressed the relationship between tau imaging ([^11^C]THK5351 and [^18^F]AV1451 binding) and CSF p-tau levels [41, 153, 166, 185–187]; there is a moderate to strong positive correlation between these biomarkers across CN subjects and varied patient groups AD (prodromal and dementia), amyloid-β-negative MCI, dementia of unclear aetiology, and several non-AD disorders (PSP, CBS, and frontotemporal dementia). When only CN individuals are examined, however, only Chhatwal et al. have shown significant associations [41, 166, 187]. Similarly, when Braak staging was used for AD patients, significant correlations were found only in those at the dementia stage (Braak III, V, and I-V combined) [166]. This may reflect greater variability in CSF p-tau levels among patients with AD dementia; indeed, many in this group showed p-tau values that, while elevated, were below the cut-off. Furthermore, although overall concordance between [^18^F]AV1451 binding and p-tau measurement was greater in AD dementia patients compared to those with prodromal AD, a higher proportion showed isolated [^18^F]AV1451 positivity [166]. This was in contrast to prodromal AD, where the opposite pattern of discordance was predominant. This finding fits with recent data showing that the symptomatic phase of AD is characterized by a decrease in CSF tau levels [188]; this may relate to a slowing in neuronal loss and/or the sequestration of p-tau in tangles [186]. In a study similar to that by Mattsson et al. [166], increased [^18^F]AV1451 binding, but not increased CSF p-tau levels, was associated with lower MMSE scores in AD patients [186], lending support to this idea. At this stage, it is unclear how discordance between tau biomarkers will be treated in the application of the A/T/N scheme. Although it has been suggested that, given the chronicity of AD, concordance will occur over time [154], longitudinal studies using serial CSF sampling are required to address this.

In the studies that addressed the diagnostic accuracy of [^18^F]AV1451 binding and CSF p-tau [185, 186], although both biomarkers performed equally well in separating prodromal AD from control subjects, [^18^F]AV1451 was superior for diagnosing AD dementia [185], suggesting that the diagnostic value of these biomarkers may vary with the disease stage. The fact that CSF p-tau did not differentiate between prodromal AD and AD dementia, while the [^18^F]AV1451 signal was higher for AD dementia, suggests that p-tau could prove to be a suitable state marker (reflecting disease intensity), while [^18^F]AV1451 could be useful as a stage marker (reflecting disease progression) [189], given that its binding  is elevated at the prodromal stage with further increases through mild-to-moderate dementia [166]. In the only study to include non-AD subjects, high agreement was found between the [^18^F]AV1451 standardized uptake value ratio and CSF p-tau measurements in differentiating the non-AD subjects from amyloid-β-positive AD patients, suggesting that these measures are largely reflective of AD-specific PHF tau pathology [186]. Given the recognition of a range of different tau fragments in the CSF characterized by differing kinetic profiles [188, 190], however, it may be that novel measures are required to ensure adequate concordance with tau imaging.

##### Neurodegeneration

In a study in CN subjects, tau and amyloid-β were found to interact; when both were increased, metabolism in the posterior cingulate cortex was decreased, which predicted AD-like memory decline [191], consistent with findings in AD [192]. In contrast, frontal hypometabolism was associated with the common age-related entorhinal tauopathy, independent of amyloid-β and without memory decline. In AD patients, studies have shown a close correspondence between [^18^F]AV1451 binding and hypometabolism across most neocortical regions [44, 65, 67, 192] and between [^18^F]THK5317 and hypometabolism, especially in frontal areas [35]. Investigation of intra-subject correlations revealed that [^18^F]AV1451 was more closely related to hypometabolism than to the MRI results or amyloid-β levels in typical and atypical AD, with the strength of the correlation positively related to global tau load and age, and thus to disease progression [193]. In AD patients, although no significant increase in [^18^F]THK5317 binding  was noted at the group level over 17 months, [^18^F]FDG binding declined in AD-related regions [121, 194], suggesting a spatiotemporal offset between tau pathology and functional changes, possibly dependent on AD severity.

The presence of tau in temporoparietal and occipital regions in CN subjects and AD patients is associated with colocalized atrophy [47, 62, 195, 196]. Local and distant correlation analyses reported negative associations between tau and MRI results. [^18^F]AV1451 binding  was most strongly associated with local atrophy in mild AD, suggesting that tau aggregation drives local neurodegeneration [46, 179]. A (nonlocal) correlation was observed between [^18^F]AV1451 in the parietal/precuneus area and MTL atrophy [196]. The relationship between local [^18^F]AV1451 binding  and antecedent longitudinal MRI changes was stronger than that between [^18^F]AV1451 uptake and cross-sectional MRI in CN individuals and AD patients [195, 196], suggesting that tau levels are most strongly related to rates of neurodegenerative change. Interestingly, recent findings [197] have shown that decreased hippocampal volume in CN subjects, an assumed proxy for tau-mediated neurodegeneration, led to reduced integrity of the hippocampal cingulum bundle; this, in turn, predicted increased tau in the posterior cingulate, as indexed by [^18^F]AV1451 binding, and worse memory.

Animal studies have indeed shown the spread of tau pathology via synaptic connections, with this process accompanied by neurodegenerative changes [198, 199]. This effect, however, was specific to amyloid-β-positive individuals. Together with previous studies [37, 38, 50] this study provides empirical support for a model in which cognitive decline occurs when tau pathology spreads beyond the MTL into unimodal and polymodal association areas, in the context of elevated cortical amyloid-β levels [200].

Data from studies in traumatic brain injury and stroke have shown that both are accompanied by a transient yet marked increase in t-tau, with no change in p-tau, with t-tau levels correlating with the severity of neuronal injury [201, 202]. Moreover, in Creutzfeldt-Jakob disease, a disorder characterized by rapidly progressive neurodegeneration in the absence of PHF tau, t-tau levels are significantly increased, with p-tau showing no or only minor changes [203, 204]. CSF t-tau is thus considered to reflect the intensity of neuronal injury at a given time point [189]. Studies that examined the relationship between [^18^F]AV1451 binding and t-tau levels [166, 185, 186] showed a moderately strong positive correlation in analyses combining CN individuals and varied patient groups, but findings were mixed among the controls (absent [41, 166] or restricted to temporal [^18^F]AV1451 binding) [187]. Among prodromal AD patients, correlations between [^18^F]AV1451 binding and t-tau levels were restricted to those with transentorhinal Braak stages while more widespread correlations were found in those with AD dementia [166].

In a combined analysis that included both prodromal AD and AD dementia patients, [^11^C]THK5351 binding in the parahippocampal gyrus was positively correlated with t-tau levels [153]. Though diagnostic performance, based on area under the receiver operating characteristic curve, was similar for t-tau and p-tau levels in AD patients vs. controls [185],

CSF tau measures were shown, in a related study, to exhibit differing patterns of association to [^18^F]AV1451: while p-tau was associated with tracer retention in temporoparietal areas, t-tau showed the strongest positive correlation with retention of [^18^F]AV1451 in prefrontal areas [186], regions known to exhibit tau pathology in more advanced stages of AD [17].

Though preliminary given the modest sample size, this finding was interpreted as supporting the idea that elevated t-tau levels are a marker of tau pathology-driven neurodegeneration. Ultimately, longitudinal studies in larger cohorts incorporating serial biomarkers (CSF and imaging) in individuals across the AD continuum will be required to properly address this question. As with p-tau and tau PET, however, future studies will need to address the interchangeability of binarized t-tau in comparison to other established measures of neurodegeneration.

Overall, the accumulation of data from studies comparing tau PET with neurodegeneration markers has provided evidence that tau PET is more closely related to neurodegeneration biomarkers (especially metabolism) than to the presence of amyloid. This knowledge is crucial for understanding which in vivo biomarkers are the most useful for measuring disease progression and which are potentially usable as outcome measures in clinical trials. The observation that tau levels and metabolism are more tightly correlated at later disease stages is also important for elucidating the disease stage at which the different biomarkers may be interchangeable. Longitudinal studies are still relatively limited, and it is critical that more longitudinal data are obtained to elucidate the effects of different tau regional distributions on downstream neurodegeneration and cognitive decline.

##### Cognitive measures

Previous research has shown an association between postmortem tau levels (NFTs, as well as more varied conformations, e.g., threads) and cognition [133, 205, 206]. A large number of in vivo studies have indicated that the binding of the first-generation tau tracers (THK family, [^18^F]AV1451, [^11^C]PBB3), in contrast to the binding of amyloid-β tracers, is associated with different cognitive measures in patients with prodromal or dementia-stage AD, in agreement with the available postmortem studies [40, 42, 44, 50, 59, 153, 166, 183, 184, 207, 208]. More importantly, performance in episodic memory tests has been associated with tracer binding loads in the temporal lobe, whereas performance in executive or global cognitive function tests was associated with binding in more widespread neocortical areas [40, 44, 183, 184, 207], a finding to be expected based on the hypothesized neuroanatomical underpinnings of specific cognitive domains. Tau tracer binding has also been associated with poorer outcomes in tests evaluating visuospatial or semantic memory, attention, language, etc. [44, 183, 208, 209]. Furthermore, declines in episodic memory performance over time appear to be associated to some extent with increases in [^18^F]THK5317 binding in the only longitudinal tau study so far examining such relationships [69]. Interestingly, there could be a relationship between [^18^F]AV1451 binding in the MTL and episodic memory performance, even in CN individuals [37, 48, 210], since underlying tau pathology might be present in these individuals, as there was subjective evidence of cognitive impairment [211].

Overall, studies have given evidence that tau is associated to global cognition as well as episodic memory. This relationship seems less strong than between metabolism and cognition, with several studies indicating that neurodegeneration mediates the effect of tau on cognition [183, 184]. The combination of these findings adds support to the proposed hypothetical sequence for the development of AD, namely the presence of amyloid-β, followed by tau, decreased metabolism, increased atrophy and cognitive decline. Less known, however, is the relationship between tau and cognition in normal aging, which suggests that the mere presence of tau (i.e., in common age-related entorhinal tauopathy) is not sufficient to cause cognitive changes [191].

##### Methodological aspects of tau PET quantification

Several tau PET studies have replicated a quantification method based on the Braak stages [37, 39, 45], but most studies use standard region-of-interest quantification. While the number of longitudinal studies is few, a recent follow-up of a large patient cohort noted that amyloid-β-positive CN individuals had showed positive neocortical tau rates beyond the expected Braak stage I/II, suggesting that tau distribution is not strictly limited to the Braak model. The authors concluded that reliable detection of within-person tau accumulation may be obtainable from simple meta-regions of interest, varying from areas showing early (posterior cingulate) and late (orbitofrontal) changes in AD, to temporal and whole brain [70]. A similar observation was made in a separate cohort [63], where elevated tau seemed to accumulate system-wide in patterns somewhat different from the expected Braak progression. These recent observations, if confirmed in future studies with postmortem validation, challenge the view that the topography of tau is more relevant to understand disease progression than a measure of global tau load [212, 213], a finding that has important clinical implications, especially in the field of clinical trials. Braak staging, and the measurement of global or region-specific tau levels, have also been shown to give similarly accurate diagnoses; however, although the whole-brain tau PET method may be an adequate AD biomarker, more localized methods in AD-vulnerable regions might increase sensitivity to early tau [214].

In contrast to a priori regionally based measures, several studies have explored data-driven methods; these include independent component analysis [38, 63, 215], cluster analyses [176, 216], hierarchical clustering [177], and graph theory [46, 181]. A cluster analysis [176] identified regions (entorhinal, amygdala, occipital, and temporal) that were most informative in discriminating between low- and high-[^18^F]AV1451 binding in CN participants. Another clustering approach [216] identified distinct patterns of entorhinal vs. neocortical [^18^F]AV1451 binding that best explained the age and phenotypical variability of typical and atypical AD patients. Further data-driven methods in new studies with second-generation tracers will be of great value for acquiring more precise knowledge of tau distribution patterns in relation to those of other biomarkers.

In summary, diverse quantification methods have been developed and applied so far in different cohorts, with sometimes conflicting results. In particular, there is accumulating evidence that the topography of tau deposition does not always follow the Braak staging scheme. Thus, other quantification methods have been applied using either a priori ROIs or data-driven approaches. Given the mentioned limited knowledge as to what individual regions may be most sensitive to early tau deposition, further studies using data-driven methods will be of great value, as these can reveal disease-specific patterns of tau deposition without any a priori assumption as to its topography. The use of highly selective second-generation tracers should facilitate the acquisition of more precise knowledge of tau distribution patterns in relation to those of other biomarkers. Finally, few studies have attempted to compare results using different quantification methods [214]; thus future studies comparing these approaches in the same cohort, as well as ante-/post-mortem comparisons, will help validate these different quantification methods, potentially leading to revised in vivo staging schemes for tau.

### Future directions

#### Temporal evolution of tau in relation to other biomarkers

Using diverse quantification methods, including data-driven approaches, new knowledge on the distribution of tau in relation to other biomarkers has been gained. While it cannot be ruled out that amyloid-β and tau pathology arise as a result of shared or independent upstream mechanisms (e.g., age or genetic related abnormalities in amyloid-β turnover/clearance [217, 218], or cardiac and metabolic conditions [219]), the evidence so far lends support to the modified amyloid-β cascade pathway, whereby amyloidosis precedes the spread of pathologic tau, which in turn causes neurodegeneration and cognitive decline [154, 172]. As mentioned, however, elevated amyloid-β and tau hyperphosphorylation may arise independently and subsequently interact in a synergistic fashion [220–223]. Specifically, it has been proposed that tau pathology may precede amyloid-β in subcortical and MTL areas, with cortical amyloid-β subsequently mediating the extra-temporal spread of tau [171], a model for which preliminary in vivo data is supportive [46, 224]. In either case, tau imaging will prove crucial to clarify the link and temporal lag between amyloid-β, tau, and neurodegeneration, and how the latter processes in particular relate to cognitive impairment.

#### Are we ready for clinical trials with tau PET?

The various stages in the maturation of tau pathology offer targets for intervention; these range from inhibiting the expression of tau itself [225, 226], to preventing post-translational modifications [227, 228] and tau aggregation [229, 230]. At present, however, most anti-tau clinical trials involve tau immunotherapy [231]. Beyond its use for patient selection and proof of target engagement [178], tau PET may prove valuable as an outcome measure [232]. In the largest longitudinal analysis performed to date on tau PET [70, 233], significant tau accumulation was shown over 1 year in clinically unimpaired amyloid-β-positive individuals, specifically, in regions shown to be the earliest sites of amyloid-β accumulation [234]. Although much validation work remains, including optimization and standardization of methodological aspects, findings from this study suggest that tau PET may be suitable for incorporation into clinical trials with individuals across the spectrum of AD [233]. Given the likely need for combinatorial approaches combining both anti-tau and anti-amyloid-β approaches [178], additional longitudinal studies are needed to elucidate the time lag between amyloid-β and tau deposition to better understand the optimal starting point for such therapies [235].

#### Potential applications of tau PET

The fast-developing research on tau PET over the past few years has promoted discussion on its usefulness for the diagnosis of neurodegenerative diseases. Although the evidence so far indicates that the currently available tracers consistently bind to PHF AD-type tau, with variable results when it comes to binding in non-AD tauopathies, tau PET seems to be a promising tool for the differential diagnosis of different tauopathies and in the work-up of atypical AD phenotypes that are difficult to diagnose. Given the seemingly reduced off-target striatal retention of second-generation compounds, studies using these ligands are likely to prove of value in the characterization of these disorders, if the tracers bind to non-AD tau-positive deposits. Studies addressing the clinical impact of tau imaging in terms of its ability to help resolve diagnostically uncertain cases, for instance, and its use in comparison to and with other established biomarkers, are critical [236]. Whether a subset of tracers will ultimately prove interchangeable, however, similar to amyloid-β ligands, or whether varied compounds will be required to cover the spectrum of tauopathies, remains an open question. More broadly, it is also expected that tau imaging could help with disease staging and to address unanswered questions pertaining to how amyloid-β and tau, both independently and via synergistic interactions, result in cognitive decline, and their relationships with other pathologies such as TDP-43 and α-synuclein, when tracers for these targets become available.
